# Identification of a novel inactivating mutation in Isocitrate Dehydrogenase 1 (IDH1-R314C) in a high grade astrocytoma

**DOI:** 10.1038/srep30486

**Published:** 2016-07-27

**Authors:** Sanne A. M. van Lith, Anna C. Navis, Krissie Lenting, Kiek Verrijp, Jan T. G. Schepens, Wiljan J. A. J. Hendriks, Nil A. Schubert, Hanka Venselaar, Ron A. Wevers, Arno van Rooij, Pieter Wesseling, Remco J. Molenaar, Cornelis J. F. van Noorden, Stefan Pusch, Bastiaan Tops, William P. J. Leenders

**Affiliations:** 1 Department of Pathology, Radboudumc, Nijmegen, The Netherlands; 2 Department of Cell Biology, Radboud Institute for Molecular Life Sciences, The Netherlands; 3 Centre for Molecular and Biomolecular Informatics, Radboud Institute for Molecular Life Sciences, The Netherlands; 4 Translational Metabolic Laboratory, Department Laboratory Medicine, Radboudumc, Nijmegen, The Netherlands; 5 Department of Pathology, VU University Medical Center, Amsterdam, The Netherlands; 6 Department of Cell Biology and Histology, Academic Medical Center, Amsterdam, The Netherlands; 7 Clinical Cooperation Unit Neuropathology, German Cancer Center (DKFZ), Heidelberg, Germany

## Abstract

The majority of low-grade and secondary high-grade gliomas carry heterozygous hotspot mutations in cytosolic isocitrate dehydrogenase 1 (*IDH1*) or the mitochondrial variant *IDH2*. These mutations mostly involve Arg132 in IDH1, and Arg172 or Arg140 in IDH2. Whereas IDHs convert isocitrate to alpha-ketoglutarate (α-KG) with simultaneous reduction of NADP^+^ to NADPH, these IDH mutants reduce α-KG to D-2-hydroxyglutarate (D-2-HG) while oxidizing NADPH. D-2-HG is a proposed oncometabolite, acting via competitive inhibition of α-KG-dependent enzymes that are involved in metabolism and epigenetic regulation. However, much less is known about the implications of the metabolic stress, imposed by decreased α-KG and NADPH production, for tumor biology. We here present a novel heterozygous *IDH1* mutation, *IDH1*^*R314C*^, which was identified by targeted next generation sequencing of a high grade glioma from which a mouse xenograft model and a cell line were generated. IDH1^R314C^ lacks isocitrate-to-α-KG conversion activity due to reduced affinity for NADP^+^, and differs from the IDH1^R132^ mutants in that it does not produce D-2-HG. Because IDH1^R314C^ is defective in producing α-KG and NADPH, without concomitant production of the D-2-HG, it represents a valuable tool to study the effects of IDH1-dysfunction on cellular metabolism in the absence of this oncometabolite.

Diffuse gliomas are primary tumors of the brain that are thought to develop from (precursors of) astrocytes or oligodendrocytes. Based on histopathology and according to WHO guidelines, these cancers are classified as grade II-IV astrocytomas or grade II-III oligodendrogliomas^1^,^2^. The disease is essentially incurable and median overall survival of patients with grade IV astrocytoma (glioblastoma, the most malignant variant) is only 15 months, despite chemo- and radiotherapy^3^.

In 2008, somatic mutations in the isocitrate dehydrogenase genes *IDH1* and *IDH2* were identified in the large majority of low grade gliomas and secondary glioblastomas^4^. These mutations are always heterozygous and mostly involve the same amino acid residues; Arg132 in the cytosolic IDH1 and Arg172 or Arg140 in the mitochondrial IDH2^5^,^6^,^7^. In glioma, the majority of *IDH* mutations are found in *IDH1*, and about 90% are *IDH1*^*R132H*^. Extensive research has revealed that these mutations are neomorphic gain-of-function mutations, resulting in the conversion of α-ketoglutarate (α-KG), the normal product of IDH-mediated decarboxylation of isocitrate, to D-2-hydroxyglutarate (D-2-HG) which can accumulate to millimolar concentrations^8^. During the conversion of α-KG to D-2-HG by the mutant enzyme, NADPH is oxidized.

Because of the molecular similarity between α-KG and D-2-HG, the latter can competitively inhibit the activity of a range of α-KG-dependent dioxygenases, of which over 60 have been identified^9^,^10^. Most important examples are the Ten-Eleven-Translocation 2 (TET2) DNA demethylases (resulting in the CpG-island hypermethylator phenotype in *IDH*^*MUT*^ cells) and Jumonji-C (JMJC) histone demethylases (resulting in histone hypermethylation)^11^,^12^,^13^. The net result is a transcriptional profile that contributes to tumorigenesis via mechanisms that are still poorly understood. Although this has been matter of debate, consensus is now that D-2-HG can promote degradation of the transcription factor hypoxia-inducible factor (HIF-1α), possibly after non-enzymatic oxidation to α-KG^14^. How this exactly contributes to oncogenesis is still an active field of investigation^15^,^16^,^17^.

The effects of expression of IDH1-mutants and/or D-2-HG on tumor metabolism have started to receive attention now^18^. It was recently shown that D-2-HG inhibits the tricarboxylic acid (TCA) cycle enzyme succinate dehydrogenase, possibly leading to hypersuccinylation of proteins and anti-apoptotic effects^19^. Because mutant IDH1 lacks isocitrate-to-α-KG conversion activity, normally a predominant source of cytosolic NADPH in the brain^20^, *IDH*^*MUT*^ cells are predicted to have lower steady state levels of NADPH, an effect that will be augmented by NADPH-oxidation during D-2-HG production. Thus, *IDH* mutations impact the redox status of glioma cells.

Additionally, *IDH* mutations may affect anabolic pathways: IDH1/2 can reduce α-KG back to isocitrate which may serve as carbon source for fatty acid and lipid synthesis via citrate and acetyl-CoA^21^,^22^ but IDH1^R132H^ lacks this reverse activity^23^. It is conceivable therefore, that *IDH*^*MUT*^ tumors need metabolic salvage pathways to allow tumor progression and this is supported by the finding of relatively normal α-KG levels in a patient-derived orthotopic *IDH1*^*R132H*^ oligodendroglioma model^24^. Because *IDH1*^*MUT*^ cells display higher sensitivity to glutaminase inhibitors than *IDH*^*WT*^ cells^25^, glutamine has been proposed to feed in into the mitochondrial TCA cycle as an anaplerotic source of α-KG, via the activities of glutaminase and glutamate dehydrogenase (GDH). We previously postulated that gliomas may resort to direct import of glutamate, a ubiquitous neurotransmitter in brain, to allow GDH-mediated α-KG production^26^,^27^. NADH/NADPH, generated during this reaction would simultaneously supplement the NAD(P)H pool. These metabolic changes could all play a role in tumor cell maintenance and therefore be an Achilles heel and target for therapeutic inhibition. However, uncoupling the metabolic alterations that result from NADPH/α-KG depletion from the pleiotrophic effects of D-2-HG is a difficult task.

In this study we describe a novel heterozygous *IDH1* mutation which we uncovered by next generation sequencing of a glioblastoma from which a patient-derived xenograft model and corresponding cell line were generated. We show that this IDH1^R314C^ mutant does not convert isocitrate to α-KG, unless at non-physiological concentrations of NADP^+^, and does not produce D-2-HG. These properties make that IDH1^R314C^ tumor models are valuable tools to study the relevance of α-KG/NADPH depletion versus D-2-HG formation in gliomagenesis and tumor progression.

## Results

### E98 cells contain a rare heterozygous IDH1^R314C^ mutation located in the NADP^+^ binding pocket

**Figure 1:**
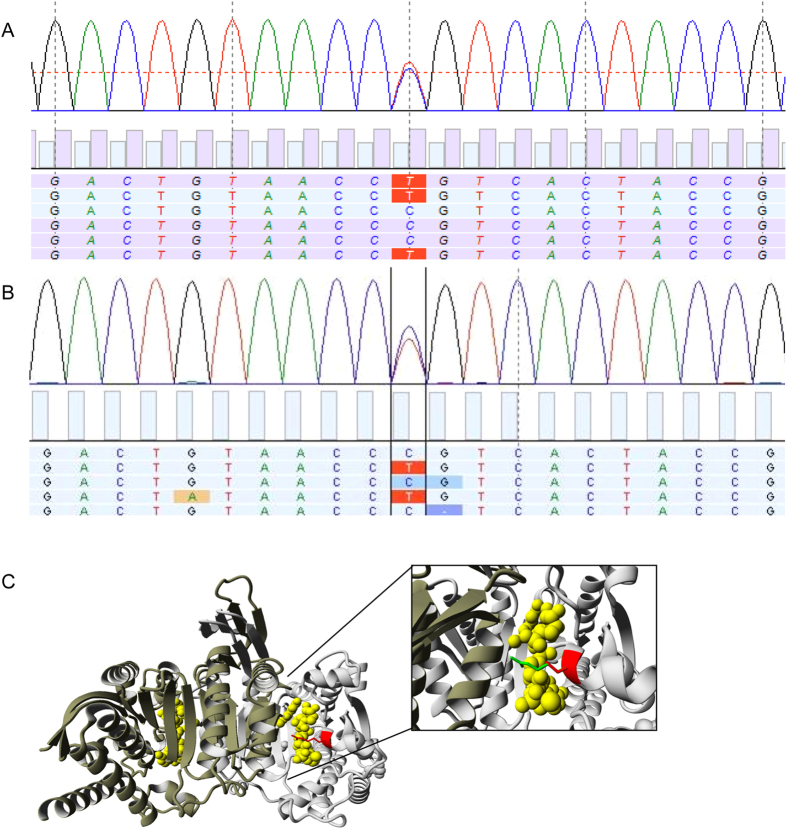
Identification of IDH1^R314C^ in a high grade astrocytoma. (**A**) Sequencing trace showing the heterozygous C to T mutation at position 940 in the *IDH1* gene found in the E98 cell line and (**B**) primary tumor material. This mutation leads to the p.Arg314Cys substitution in the IDH1 protein. (**C**) Display of the 3D- structure of human IDH1^R314C^ generated with YASARA (PDB-file 3inm). The IDH1^WT^ homodimer is displayed with bound cofactor NADP^+^ (yellow), and the arginine residue on position 314 (red), both subunits are coloured in a different shade of grey. The inset shows the mutated cysteine (red) superimposed on the original arginine (green).

The patient-derived E98 astrocytoma model carries a number of glioma-typical genetic mutations and is phenotypically similar to clinical glioma when grown as orthotopic xenografts^28^,^29^,^30^. This makes this model of high interest as a prototypical glioblastoma model for testing of targeted therapeutics^31^,^32^,^33^,^34^. Because the design of rational targeted therapies requires a detailed analysis of genetic aberrations, we subjected this cell line to targeted genomic next generation sequencing via Ion Torrent analysis using a primer set that allows parallel deep sequencing of 409 genes with known involvement in cancer-related pathways. As we unfortunately did not have access to blood of the E98 donor, SNPs and variants with mean allelic frequency (MAF) > 1% were filtered out using sequence data from pooled blood samples as reference. Due to our general interest in *IDH1*, our attention was specifically drawn to a heterozygous C to T mutation at position 940 in the *IDH1* gene (Fig. 1A) which results in the p.Arg314Cys substitution. Presence of this mutation was verified in the original patient material (Fig. 1B). To check for the incidence of this mutation, 103 DNA samples from glioma (see Supplementary Table 1) were Sanger sequenced. None of these samples contained the p.R314 mutation. Data mining of the Exome Sequencing Project (ESP) database (www.exac.broadinstitute.org/) revealed that this variant has not been identified in 121,410 alleles. Mining of the Cosmic database revealed only one reported IDH1 c.941G>A/p.R314H mutation in a gastric carcinoma (mutation ID COSM4090677). Thus, the R314C mutation is a rare mutation.

Since Arg314 is located in the NADP^+^ binding pocket^35^ we studied the experimentally solved structure of IDH1^WT^ and IDH1^R314C^ in complex with NADP^+^ using YASARA. As shown in Fig. 1C, the R314C mutation significantly alters the tertiary structure resulting in an imperfect fit of NADP^+^ in the binding pocket, mainly due to the lack of interaction between the negatively charged 2′-phosphate of the co-enzyme NADP^+^ and the positively charged amine group of the arginine, and lack of ionic interactions between the Arg314 and glutamate residues on position 247 and 253 in the other subunit.

### IDH1^R314C^ is defective in isocitrate to α-KG conversion

**Figure 2:**
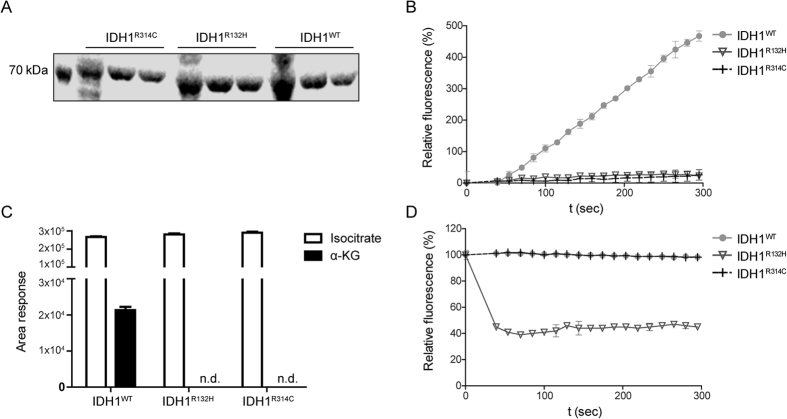
Homodimeric IDH1^R314C^-GST is defective in the isocitrate-to α-KG reaction and does not produce D-2-HG. (**A**) SDS-page gel stained with coomassie brilliant blue showing expression of purified IDH1-GST constructs. (**B**) Fluorescent monitoring of NADPH formation at 340 nm shows that only purified IDH1^WT^-GST is capable to convert isocitrate to α-KG under the reaction conditions tested, whereas IDH1^R132H^-GST and IDH1^R314C^-GST are inactive. (**C**) α-KG production by IDH1-GST enzymes was measured with LC-MS. Note that only IDH1^WT^-GST is capable of isocitrate-to-α-KG conversion. (n.d. = non detectable, area < 150). (**D**) Fluorescent monitoring of NADPH reduction shows that only purified IDH1^R132H^-GST is active in the α-KG-to-D-2-HG conversion. Both IDH1^WT^-GST and IDH1^R314C^-GST lack the capability to produce D-2-HG.

We first examined the activity of purified IDH1^R314C^-GST proteins, using IDH1^WT^-GST and IDH1^R132H^-GST as positive and negative controls. All enzymes could be readily produced and purified (Fig. 2A). Under the conditions tested, both IDH1^R132H^-GST and IDH1^R314C^-GST did not show isocitrate-to-α-KG conversion activity whereas IDH1^WT^-GST did (Fig. 2B). The absence of α-KG formation by IDH1^R132H^-GST and IDH1^R314C^ was confirmed with LC-MS (Fig. 2C). Importantly, whereas IDH1^R132H^-GST was active in converting α-KG to D-2-HG (as revealed by oxidation of NADPH) the IDH1^R314C^-GST mutant was completely devoid of this activity (Fig. 2D). Thus, IDH1^R314C^ homodimers are inactive in converting isocitrate and NADP^+^ to α-KG and NADPH and, unlike IDH1^R132H^-GST, do not consume NADPH, consistent with the lack of D-2-HG production by E98 xenografts that has already been described before^36^.

**Figure 3:**
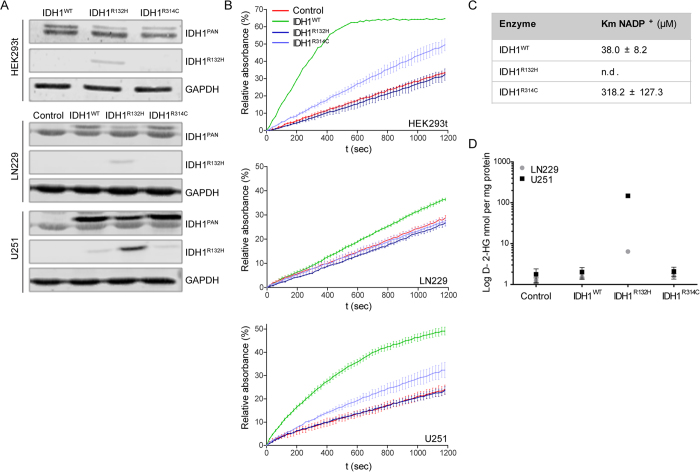
IDH1^R314C^-BAPHIS expression in glioma cell lines leads to reduced forward activity compared to IDH^WT^ expression. (**A**) Western blots showing expression of IDH1-BAPHIS in transiently transfected HEK293t and lentivirally transduced LN229 and U251 glioma cells. Blots were stained with anti-IDH1^PAN^ and anti-IDH1^R132H^ as indicated, or with anti-GAPDH as a loading control. Since the recombinant proteins are ~3 kDa larger than endogenous IDH1 they can be distinguished from the endogenous IDH1. Control LN229 and U251 were transduced with empty vector virus (EV). (**B**) Monitoring of NADPH formation after isocitrate/NADP^+^ addition to extracts from transfected/transduced cell lines. NADPH formation was monitored by absorbance measurements at 340 nm. Note that IDH1^R314C^-BAPHIS was far less effective in oxidative carboxylation of isocitrate than IDH1^WT^-BAPHIS. (**C**) Km values of IDH1-GST for NADP^+^. The Km value for NADP^+^ for IDH1^R132H^-GST could not be determined. (**D**) D-2-HG production by IDH1-BAPHIS expressing cell lines was measured with LC-MS. Only IDH1^R132H^-BAPHIS expressing cells were capable of D-2-HG production.

The IDH1^R132^ mutation in human gliomas always occurs in a heterozygous manner, resulting in a theoretical 1:2:1 distribution of IDH^WT/WT^, IDH^WT/MUT^ and IDH^MUT/MUT^ dimers. This stoichiometry may be important for balanced IDH activity, because D-2-HG can only be formed via an intimate crosstalk between wild-type and mutant subunits, for which α-KG is a product and substrate, respectively. To analyze the effects of IDH1^R314C^ overexpression in eukaryotic cells we performed enzymatic assays with cell extracts from HEK293T cells, transiently overexpressing IDH1^WT^, IDH1^R132H^ or IDH1^R314C^ as well as lentivirally transduced LN229 and U251 glioma cells, stably expressing the different IDH1 mutants (Fig. 3A). To be able to distinguish recombinant from endogenous IDH^WT^ and allow statements on IDH^WT/MUT^ ratios we equipped the mutated reading frames with a BAPHIS (biotin-acceptor peptide-8xHis) tag, increasing molecular weight with ~3 kDa. Intriguingly, irrespective of the method of genetic modification (transfection or lentiviral transduction) expression levels of recombinant enzymes were lower than that of the endogenous enzymes, with IDH^R132H^ consistently being lowest, possibly a result of negative selection for IDH1^R132H^-BAPHIS overexpression and associated toxic levels of D-2-HG^37^. Consistent with the absence of activity of purified IDH1^R314C^ homodimers (Fig. 2B), for all cell lines NADP^+^-dependent oxidative decarboxylation of isocitrate was significantly lower in IDH1^R314C^-BAPHIS expressing cells than in IDH1^WT^-BAPHIS cells (Fig. 3B). Of note, the reduced activity of IDH1^R314C^-BAPHIS in U251 cell extracts could be rescued by performing the enzymatic assay in supraphysiological concentrations of NADP^+^ (Supplementary Figure 1), in line with loss of NADP^+^-affinity as predicted from the solved structure in Yasara, and as described earlier by Lee *et al.*^35^. Consistent with this model, the R314C mutation caused an eight-fold increase in Km for NADP^+^ (318 μM vs 38 μM for IDH^WT^, Fig. 3C). This predicts that IDH1^R314C^ is inactive under physiological NADP^+^ concentrations. Of note, activity of GST-IDH1^R314C^ homodimers at higher concentrations of NADP^+^ was too low to calculate Km for isocitrate.

As expected from the lack of NADPH consumption when incubated with α-KG (Fig. 2D), IDH1^R314C^-BAPHIS expressing LN229 and U251 glioblastoma cell lines did not produce 2-HG, in contrast to their IDH1^R132H^-BAPHIS counterparts (Fig. 3D).

## Discussion

Since their discovery, hotspot mutations in IDH enzymes have become a major focus of research. The oncogenic potential of IDH1^R132H^ has been attributed to epigenetic alterations, resulting from the production of the alleged oncometabolite D-2-HG^9^,^10^,^38^. Furthermore it has been shown that proliferation of *IDH1*^*R132H*^ cells requires metabolic rescue programs that depend on *in vivo* tumor-stroma interactions, possibly involving glutamate import^26^.

We here identify and characterize IDH1^R314C^, a novel and rare mutation in IDH1 in a high grade astrocytoma which has not been reported before. We show that IDH1^R314C^ has a greatly reduced affinity for NADP^+^, in agreement with a previous structure-function relationship study on porcine IDH1^35^ and is consequently defective in oxidizing isocitrate to α-KG. The R314C mutation did not shift the cofactor dependency from NADP^+^ to NAD^+^ (Supplementary Figure 2) as was previously reported for mutated IDH1 from the halobacterium *Haloferax Volcanii*^39^.

To define an exact role for IDH1^R314C^ in tumor formation and biological behavior of cancer cells is impossible in the context of the many more mutations that are found in E98^40^. To study this further, isogenic E98 cell lines with a repaired IDH1^R314R^ allele are required and such technically challenging cell lines are not yet available.

The decreased potential of *IDH1*^*R314C*^ cells to produce α-KG and NADPH in the cytosol may directly impact on redox potential, lipid synthesis and membrane production and may explain the slow growth rates of E98 cells *in vitro* (doubling time of 46 hrs, data not shown). Interestingly, despite these very slow growth rates, *in vivo* E98 xenografts represent the most aggressive diffuse infiltrative brain tumor model in our lab, killing mice within 4–5 weeks after intracerebral implantation^28^. This illustrates that the brain micro-environment is crucially involved in allowing growth of E98 cells. Since the redox status of cells may particularly impact on cell migration^41^ it would be of high interest to investigate how relatively low NADPH/NADP^+^ ratios in E98 cells *in vivo* are associated with active cell migration, leading to the diffuse infiltrative phenotype. Further unraveling of the specific *in vivo* metabolic salvage pathways that are adapted by E98 cells and may be involved in its aggressive behavior may lead to potential novel ways for therapeutic intervention.

In oncology, most NGS protocols aim for detection of actionable hot spot mutations in known genes only, leaving a potentially huge amount of mutations with significance for tumor biology, undetected and this is the reason why we only now recognized that E98 cells carry the IDH1^R314C^ mutation^24^. Alterations in genes encoding metabolic enzymes may be directly or indirectly involved in tumorigenesis based on subtle distortions in equilibria between metabolic intermediates and/or oxidative milieu.

In conclusion, we identified a novel IDH1^R314C^ mutant that shares with the common IDH1^R132H^ mutant a defect in isocitrate-to-α-KG conversion activity, but lacks the capacity to produce D-2-HG. Future comparative profiling studies using isogenic cell lines expressing IDH^WT^, IDH1^R132H^ or IDH1^R314C^ will allow us to distinguish between epigenetic and metabolic alterations, resulting from NADPH/α-KG depletion or D-2-HG accumulation.

## Methods

### E98

Generation of the E98 model which was derived from a grade IV astrocytoma, was described before^28^. All animal experimental work was performed in accordance with the guidelines of and was approved by the local ethical committee for animal experimentation of Radboud University. An adherent E98 cell line was generated from mouse xenografts via routine procedures^29^,^40^ and cultured in DMEM (Lonza, Basel, Switzerland) supplemented with 10% FCS (Gibco, ThermoFisher Scientific, Watlham, MA, USA) and 40 μg/ml gentamycin (Centrafarm, Etten-Leur The Netherlands).

### Next generation sequencing

Genomic DNA from E98 cells and from original tumor material was analyzed by semi-conductor sequencing (IonPGM, Life Technologies, ThermoFisher Scientific, Watlham, MA, USA) using the comprehensive cancer panel (Life Technologies, ThermoFisher Scientific, Waltham, MA, USA) detecting copy number variations and mutations in 409 cancer-related genes. An IonPGM E98 library was generated according to the manufacturer’s protocol. In short, 10 ng of DNA per pool was amplified in 21 cycles by PCR using the Ion AmpliSeqTM mastermix, followed by barcode and adapter ligation. Amplified products were purified with Agencourt AMPure XP beads (Beckman Coulter Genomics, High Wycombe, UK). The library was diluted to 20 pM and emulsion PCR was performed using the Ion OneTouchTM 200 Template kit following the manufacturer’s protocol. Next, Ion Sphere Particles (ISPs) were recovered and enriched for template positive ISPs using Dynabeads MyOne Streptavidin C1 beads (Life Technologies, ThermoFisher Scientific, Waltham, MA, USA) in the Ion OneTouchTM ES instrument (Life Technologies, ThermoFisher Scientific, Waltham, MA USA). ISP enrichment was quantified using the Qubit 2.0 fluorometer (Life Technologies, ThermoFisher Scientific, MA, USA). Sequencing primer and polymerase were added to the final enriched spheres before loading onto an Ion 318 chip according to the Ion PGMTM 200 sequencing kit protocol. Variants were called using NextGene (JSI) and filtered for common variants (MAF > 1%) in the general population.

### Visualization of IDH1 structure with YASARA

The structure of human IDH1 has been experimentally solved and can be found in PDB-file 3inm^8^. We used the WHAT IF & YASARA twinset for visualization and analysis^42^,^43^.

### PCR and cloning of IDH1 constructs

Total RNA was isolated from cell lines using TRIzol reagent (Life Technologies, ThermoFisher Scientific, Waltham, MA, USA) and converted to cDNA using oligo-dT and MMLV reverse transcriptase (New England Biolabs, Ipswich, MA, USA) using standard protocols. Open reading frames for *IDH1*^*WT*^, *IDH1*^*R132H*^ and *IDH1*^*R314C*^ were PCR amplified from HT29, E478 and E98 cDNA, respectively, using primers IDH-EcoRIFw [5′-CGAATTCACTGTCAAGGTTTATTGAAGTC-3′] and IDH-AgeIRev [5′-CACCGGTAAGTTTGGCCTGAGCTAG-3′] and Phusion DNA polymerase (Finnzymes, ThermoFisher Scientific, Watlham, MA, USA). PCR products were digested with EcoRI and AgeI (New England Biolabs, Ipswich, MA, USA) and cloned in fusion to a C-terminal biotin-acceptor peptide (BAP) and an 8xhis tag (HIS) in vector PHLsec-BAPHIS AgeI/EcoRI, derived from PHLsec-HIS (Addgene, Cambridge, MA, USA) using T4 DNA ligase (New England Biolabs, Ipswich, MA, USA). The BAPHIS tags were added to distinguish recombinant from endogenous IDH by molecular weight and to allow Ni-bead purification. Clones containing the respective mutations were selected via Sanger sequencing. For generation of lentiviral constructs, the different ORFs were recloned in vector pENTRY which was recombined with pLENTI using the GATEWAY system via standard procedures.

In parallel, IDH ORFs were cloned in fusion with GST in *E. coli* expression vector pDEST15 as described before^37^.

### GST fusion protein expression and purification

GST fusion proteins were expressed in *E. coli* strain ER2566 via standard induction protocols. Briefly, 2xTY medium supplemented with 50 μg/ml ampicillin (Roche, Basel, Switzerland) was inoculated with 1% v/v overnight culture. Protein expression was induced with 1 mM IPTG (Serva, Heidelberg, Germany) at OD_600_~0.6. After 3 hours at 30 °C, bacteria were harvested by centrifugation and resuspended in 300 mM NaCl, 50 mM TRIS/HCL pH 8.0 supplemented with Complete protease inhibitor (Roche, Basel, Switzerland). Cells were lysed by 3 freeze-thaw cycles, followed by sonication (4 × 15 seconds, 25% amplitude, Bioruptor, Diagenode) and centrifugation (3500 rpm, 20 min). Proteins were purified using glutathione beads (Pierce, ThermoFisher Scientific, Waltham, MA, USA), eluted with 10 mM glutathione and dialyzed extensively against 150 mM NaCl, 25 mM TRIS/HCL pH 7.5. Expression and purity were checked by SDS-PAGE gel electrophoresis and Coommassie Brilliant Blue staining.

### Transient transfection experiments

HEK293T cells were grown to 30% confluence in 6 wells plates in DMEM supplemented with 10% FCS and 40 μg/ml gentamycin. Cells were transfected with 2 μg pHLsec-IDH1^MUT^-BAPHIS, IDH1^WT^-BAPHIS or pIRESneo-EGFP using Fugene HD reagent according to the manufacturers’ protocol (Roche, Basel, Switzerland). After 2 days, cells were harvested and processed to cell lysates by scraping in 150 μl sucrose buffer (0.32 M sucrose in 10 mM TRIS-HCL pH 7.5) and sonicating on ice (3 cycles of 30 sec max power and 30 sec off in Bioruptor, Diagenode). Lysates were centrifuged (14000 rpm, 10 min, 4 °C) and supernatants were subjected to BCA assays (Pierce, ThermoFisher Scientific, Waltham, MA, USA) for protein concentration measurements and stored at −20 °C until further analysis. To quantify IDH1 protein expression, 20 μg of total cytosolic protein was subjected to 10% SDS-PAGE and electroblotted onto a nitrocellulose membrane (Whatman Optitran BA-S85, GE healthcare, Little Chalfont, UK). After blocking in Odyssey Blocking buffer (LI-COR biosciences, Licoln, NE, USA) in PBS (1:1) the membrane was incubated with rabbit-anti-IDH1 (1:1000, HPA035248, Sigma-Aldrich, St. Louis, MO, USA) and mouse-anti-IDH1R132H (1:400, H09L, Dianova, Hamburg, Germany) in blocking buffer, followed by incubation with goat-anti-mouseDyLight800 (1:10.000, ThermoFisher Scientific, Waltham, MA USA) or goat-anti-rabbitAlexa680 (1:10.000, Invitrogen, ThermoFisher Scientific, Waltham, MA, USA). After washing, blots were analyzed on the Odyssey scanner (LI-COR biotechnology, Lincoln, NE, USA).

### Generation of stable IDH1^MUT^ overexpression glioma cell lines

LN229 and U251 cells were transduced with lentiviral vectors encoding IDH1^WT^-BAPHIS, IDH1^R132H^-BAPHIS, IDH1^R314C^-BAPHIS or an empty vector (EV) control as described before^44^. Transduced cells were selected using blasticidin and cultured in DMEM supplemented with 10% FCS and 40 μg/ml gentamycin. Cells were grown to 80% confluency in 10 cm ø culture plates (Corning, Corning, NY, USA). Cell culture media were collected and stored at −20 °C until later analysis. Cells were collected and cytosolic extracts were prepared in 400 μl sucrosebuffer as described before. Twenty μg of protein was subjected to SDS-PAGE gel electrophoresis and western blot to quantify protein expression.

### IDH activity measurements

Isocitrate-to-α-KG and α-KG-to-2-HG conversion rates of the purified IDH1-GST enzymes were determined by monitoring NADPH generation over time. Assays were performed in 100 μl freshly prepared assay solution (2 mM MgCl_2_ in 100 mM TRIS/HCL pH 7.4) containing 10 mM NADP^+^ (Sigma-Aldrich, St. Louis, MO, USA) and 10 mM isocitrate (Sigma-Aldrich, St. Louis, MO, USA) (forward assay) or 10 mM NADPH (Sigma-Aldrich, St. Louis, MO, USA) and 10 mM α-KG (Sigma-Aldrich, St. Louis, MO, USA) (reverse assay). Four μg of purified IDH1-GST fusion proteins were incubated with assay solution in a 96 wells plate at 37 °C (BD Falcon, Franklin Lakes, USA) and fluorescence (Ex. 340 +/− 10 nm, Em. 440 +/− 10 nm), was measured every 15 seconds over a period of 5 minutes with an Omega FluoStar (BMG Labtech, Ortenberg, Germany). To examine enzyme kinetics in more detail, 4 μg of IDH1-GST proteins were incubated with varying NADP^+^ concentrations (0–500 μM) and 100 μM isocitrate at 22 °C. Absorbance at 340 nm was measured every 20 seconds with a Benchmark Plus Microplate Spectrophotometer (Bio-Rad, Hercules, CA, USA). Non-linear regression analysis (Michaelis-Menten) was performed in Graphpad Prism 5.03.

To determine NAD^+^ dependency, 4 μg of IDH1-GST proteins were incubated with 100 μl assay solution containing 2 mM MgCl_2_, 500 μM isocitrate and 2, 5 mM NAD^+^ (Sigma-Aldrich, St. Louis, MO, USA) in 100 mM TRIS-HCL pH 7.4 at 22 °C. A sucrose extract made from human liver was taken as a control. Absorbance at 340 nm was monitored as described.

For enzyme measurements in extracts of eukaryotic cells, 50 μg of total cytosolic protein were incubated with assay solution (2 mM MgCl_2_, 500 μM isocitrate in 100 mM TRIS/HCL pH 7.4) containing 100 μM NADP^+^ in 96 wells plates (Greiner) at 22 °C and NADPH generation was quantified as described before.

### D-2-HG, α-KG and isocitrate measurements by isotope dilution LC-MS

D-2-HG levels in cell culture media were measured using stable isotope dilution liquid chromatography tandem mass spectrometry (LC-MS) as described before^36^.

α-KG and isocitrate levels were determined in *in vitro* IDH1 reaction mixtures. 4 μg of IDH1-GST proteins were incubated in 150 μl freshly prepared assay solution (2 mM MgCl_2_ in 100 mM TRIS/HCL pH 7.4) containing 500 μM isocitrate and 100 μM NADP^+^. After 20 min incubation at 22 °C the reaction was stopped using 20 μl 4% HCOOH. 25 μl of this solution was mixed with 25 μl 9 μM ^13^C_5_-2-HG stable isotope (Chiralex, Nijmegen, The Netherlands) in deionized water and 75 μl deionized water before passing it through an Amicon Ultra 30 kDa Centrifugal Filter (Merck Millipore, Billerica, MA, USA) by centrifugation (14000 g, 30 min). 2 μl of the filtrate was injected onto a Atlantis T3 HPLC Column (2.1*100 mM dp 3 μ) and run with 12.5% MeOH in 87.5% 0.3% HCOOH in water. The column was connected to a Waters Quattro Premier tandem mass spectrometry fitted with an ESI probe operated in negative mode, recording the following MRM transitions, as summarized in Table 1.

### Statistics

All experiments were performed in triplicate. Data were processed in Excell (Microsoft, Redmond, WA, USA) and enzyme kinetics were determined by fitting of Michaelis Menten in Graphpad Prism 5.03 (Graphpad Software, La Jolla, CA, USA).

## Additional Information

**How to cite this article**: van Lith, S. A. M. *et al.* Identification of a novel inactivating mutation in Isocitrate Dehydrogenase 1 (IDH1-R314C) in a high grade astrocytoma. *Sci. Rep.* **6**, 30486; doi: 10.1038/srep30486 (2016).

## Supplementary Material

Supplementary Information

## Figures and Tables

**Table 1:** MRM transitions for metabolite detection.

MRM	Compound name	cone V (V)	Coll eV (eV)	Ret. time (min)
191 > 111	Isocitrate	22	13	1.28
145 > 101	α-KG	22	8	1.53
147 > 129	D-2-HG	19	11	1.54
152 > 134	^13^C_5_-2-HG	19	11	1.54

note: t0 = 0.8 min.
