# Supplementary material for: Identification of a novel inactivating mutation in Isocitrate Dehydrogenase 1 (IDH1-R314C) in a high grade astrocytoma
Source: Sci Rep. 2016 Jul 27;6:30486. doi: 10.1038/srep30486 (PMC4962051; doi:10.1038/srep30486)
Supplement: Supplementary Information [file srep30486-s1.pdf]

Supplementary data to 'Identification of a novel inactivating mutation in Isocitrate Dehydrogenase 1 (IDH1-R314C) in a high grade astrocytoma.'

Sanne A.M. van Lith[1], Anna C. Navis[1], Krissie Lenting[1], Kiek Verrijp[1], Jan T.G. Schepens[2], Wiljan J.A.J. Hendriks[2], Nil A. Schubert[1], Hanka Venselaar[3], Ron A. Wevers[4], Arno van Rooij[4], Pieter Wesseling[1,5], Remco J. Molenaar [6], Cornelis J.F. van Noorden[6], Stefan Pusch[7], Bastiaan Tops[1] and William P.J. Leenders[1*]

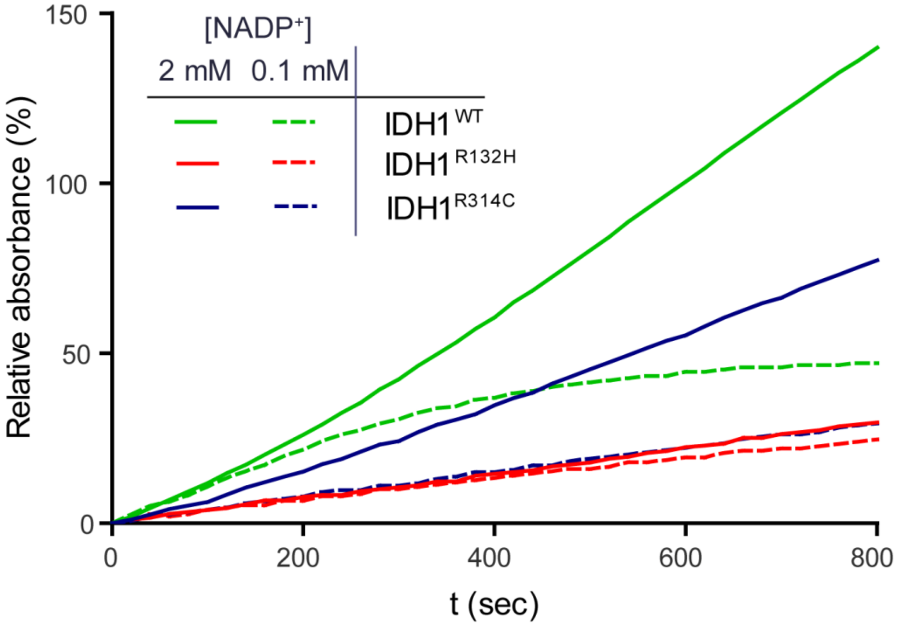


Supplementary Figure 1. IDH1$^{R314C}$ activity can be rescued by supraphysiological NADP$^{+}$ levels. Extracts of U251-IDH1$^{R314C}$ U251-IDH1$^{R132H}$ or U251-IDH1$^{WT}$ were incubated with isocitrate and 100 µM or 2 mM NADP$^{+}$, and NADPH formation was measured in real time by 340 nm absorbance. The activity of IDH1$^{R314C}$-BAPHIS but not IDH1$^{R132H}$-BAPHIS was partly rescued by increasing NADP$^{+}$ concentrations to 2 mM.

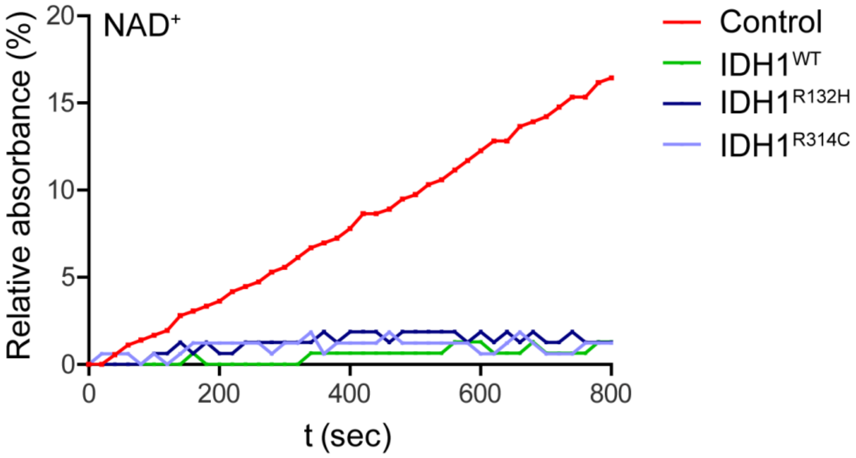


Supplementary Figure 2. The R314C mutation does not shift the cofactor dependency from $NADP^+$ to $NAD^+$. $IDH1^{MUT}$-GST enzymes were incubated with isocitrate and $NAD^+$, and NADH formation was measured by 340 nm absorbance. All IDH1 enzymes were inactive in the presence of $NAD^+$. As a positive control a liver extract was used.

| Diagnosis | Number of samples | Number of samples verified IDH1$^{R132H}$ | Number of samples verified IDH1$^{WT}$ |
|---|---|---|---|
| GBM | 47 | 1 | 42 |
| Astrocytoma grade II | 13 | 5 | 8 |
| Astrocytoma grade III | 2 | 0 | 1 |
| Oligodendroglioma grade II | 18 | 13 | 3 |
| Oligodendroglioma grade III | 3 | 0 | 2 |
| Oligodendroglioma grade IV | 1 | 0 | 1 |
| Oligoastrocytoma grade II | 14 | 9 | 3 |
| Oligoastrocytoma grade III | 3 | 0 | 3 |
| Oligoastrocytoma grade IV | 2 | 2 | 0 |

Supplementary Table 1. Overview of glioma samples used for Sanger sequencing of *IDH1*$^{R314C}$, with diagnosis and *IDH1* status.
